# Impact of Delayed Sequential Bilateral Cataract Surgery on Diabetic Macular Edema: A Real-World Study in Northwestern China

**DOI:** 10.1155/2024/2367292

**Published:** 2024-07-19

**Authors:** Yuecong Ren, Hong Yan

**Affiliations:** Shaanxi Eye Hospital Xi'an People's Hospital (Xi'an Fourth Hospital) Affiliated People's Hospital of Northwest University, Xi'an 710004, Shaanxi Province, China

## Abstract

**Objectives:**

To assess the incidence and characteristics of treatment-requiring diabetic macular edema (TR-DME) in eyes after delayed sequential bilateral cataract surgery in patients with diabetes mellitus.

**Methods:**

This was a retrospective study involving patients with diabetes who underwent cataract surgery at Shaanxi Eye Hospital between January 2019 and December 2021. The incidence and characteristics of a first episode of TR-DME after delayed sequential bilateral cataract surgery were observed.

**Results:**

A total of 1553 individuals (3106 eyes) were included in the study. All patients underwent bilateral cataract surgery with the prescribed interval of surgery within one month. The incidence of TR-DME was 0.52% in the year before surgery versus 1.87% in the year after surgery (*p* < 0.05). The highest risk period was between 2 and 4 months after surgery. A first episode of TR-DME was observed in 58 eyes of 35 patients after delayed sequential bilateral cataract surgery. The patients were divided into four groups according to the interval between bilateral cataract surgeries. A higher incidence of TR-DME was observed when the interval between the surgeries was less than two weeks.

**Conclusions:**

This is the first real-world study of the effects of cataract surgery on the development of TR-DME. The study was performed at the largest ophthalmic center in Northwestern China. The findings demonstrate that the incidence of TR-DME increased significantly after cataract surgery, with the highest incidence between two and four months after surgery. Patients with shorter intervals between bilateral cataract surgeries were at a high risk.

## 1. Introduction

Diabetic cataract (DC) is one of the main causes of visual impairment in patients with diabetes mellitus (DM). A survey on ocular complications in DM patients in Northeastern China revealed that the top cause of blindness in DM patients was diabetic retinopathy (DR), while the top cause of visual impairment in DM patients was DC [1]. Persistent high glucose levels accelerate the occurrence and development of DC via various pathways, including alterations in the osmotic pressure of the lens, the induction of oxidative stress in the lens, and increased glycosylation of lens proteins [2–4]. Indeed, cataract is significantly more common in DM patients than in the non-DM population (2–4 times in older adults) [5].

Cataract surgery is currently the only effective option for the treatment of cataracts. However, patients with DM typically have fragile intraocular structures and experience intense inflammatory reactions, with some also having diseases of the fundus and significantly higher incidences of various complications compared with the non-DM population. Diabetic macular edema (DME) is one of the main causes of DC-associated visual impairment following cataract surgery. Cataract surgery often causes or exacerbates DME due to factors such as disruption of the blood-retinal barrier due to increased levels of VEGF and other inflammatory factors. Although the impact of ultrasound use on the retina during surgery has not yet been clarified, several studies have suggested a correlation between the duration of ultrasound and DME. Yang et al. [6] analyzed the risk factors for subsequent DME development and found that changes in the macular foveal thickness were positively correlated with HbA1C or ultrasound duration. Mackenbrock et al. [7] reported similar findings, adding that there were significant associations between the macular thickness and both the cumulative dissipated energy (CDE) and ultrasound duration.

Yao et al. [8] demonstrated that compared to non-DM patients, patients with mild or moderate nonproliferative diabetic retinopathy (NPDR) had lower choroidal vascular indices, with both the choroidal vascular index and choroidal thickness increasing more rapidly after cataract surgery. Feng et al. [9] compared optimal coherence tomography angiography (OCTA) evaluations before and after cataract surgery and found that the surgery increased macular thickness in both DM and non-DM patients, as well as increasing the superficial capillary plexus of DM patients within three months of surgery.

Despite demonstrations of the effects of cataract surgery on the intraocular microenvironment, the factors influencing the occurrence of postoperative DME are not fully understood. To date, most clinical studies have focused on the development of DME following conventional cataract surgery and have not investigated the relationship between bilateral cataract surgery and DME.

The interval between bilateral sequential cataract surgeries can vary. Immediate sequential bilateral cataract surgery (ISBCS) involves the removal of cataracts from both eyes of a single patient during the same surgery, in contrast to delayed sequential bilateral cataract surgery (DSBCS) in which the cataracts are removed sequentially within a certain timespan [10]. The COVID-19 pandemic sparked a greater interest in the use of ISBCS in recent years because of the cost savings and convenience [11]. However, it is not widely performed in China due to associations with possible bilateral postoperative endophthalmitis, refractive error, and medical insurance [12]. DSBCS thus remains the preferred option for bilateral cataract surgery, as it allows for adjustment of the intraocular lens power in the second eye in the case of refractive surprise and provides time to monitor the possible development of endophthalmitis or other complications that can cause severe vision loss [13]. While the interval between surgeries is highly important in DSBCS, there is no guide or consensus in China to determine its optimal duration. In China, the interval is usually 1 to 2 weeks or more between eyes. For patients with diabetes, cataract surgery in the first eye may affect the microenvironment of the second eye, potentially inducing a greater inflammatory response [14]. However, it is not known whether different intervals influence inflammation or cause macular edema, and these are important considerations.

The purpose of this study was to investigate the incidence and characteristics of DME after bilateral sequential cataract surgery in the real world of Northwestern China, to provide a reference for the clinical diagnosis and treatment of DME.

## 2. Methods

### 2.1. Subjects

This was a retrospective study. Patients with DC who visited Xi'an People's Hospital (Xi'an Fourth Hospital) from January 2019 to December 2021 were selected, and their general information and surgical records were extracted from the electronic inpatient medical record system of Xi'an People's Hospital (Xi'an Fourth Hospital). All surgeries were performed by chief physicians and deputy chief physicians with extensive experience in phacoemulsification surgery. The study followed the requirements and guidelines of the Helsinki Declaration and was reviewed and approved by the Ethical Review Committee of Xi'an People's Hospital (Xi'an Fourth Hospital) (20220019).

### 2.2. Inclusion and Exclusion Criteria

The inclusion criteria were as follows: (1) age ≥45 years old; (2) two or more in-hospital surgical treatments; (3) cataract surgery performed by the conventional phacoemulsification + IOL implantation.

The exclusion criteria were as follows: (1) the presence of preoperative complications such as uveitis, glaucoma, and other eye diseases potentially affecting the cataract surgery; (2) serious intraoperative complications such as posterior capsule rupture, vitreous detachment, and nuclear subsidence; and (3) an interval between bilateral cataract surgeries of more than one month.

### 2.3. Assessments and Follow-Up

All patients received comprehensive ophthalmological examinations before cataract surgery. Fundus photography and optical coherence tomography (OCT) examinations were performed less than 1 month before surgery. Both OCT imaging and fundus photography were performed by skilled ophthalmic technicians. Surgeons evaluated the morphology and thickness of the macula in the OCT images and grading of the DR in the fundus photographic images; these were documented in the inpatient medical records. None of the patients had preoperative macular edema; in the event of macular edema, cataract surgery would have only been performed after treatment with standardized intravitreal anti-VEGF drugs or Ozurdex therapy.

The routine follow-up for all patients was 1 day, 1 week, 1 month, and 3 months postoperatively. At the 1- and 3-month visits, the eyes were examined with OCT and the postoperative DME was assessed. In this study, DME was defined as TR-DME, in accordance with a UK large-sample retrospective study [15]. This referred specifically to a serious type of DME that was considered to require vitreous anti-VEGF injection by the consulting physician after evaluation of the patient's visual acuity, OCT, and other imaging tests.

### 2.4. Data Collection

Patient information was retrieved from the inpatient medical record system. This information included the sex, age, and course of DM and other diseases of the patients. The presence or absence of DR and DR stage before cataract surgery, as well as any previous history of anti-VEGF injections and other surgical procedures, were recorded. Additionally, details of the surgeon and the interval of bilateral cataract surgery, history of the first anti-VEGF injection after cataract surgery, and time of presentation were recorded.

The primary observation index represented the incidence and characteristics of TR-DME in DM patients after cataract surgery. This included a comparison of the characteristics of patients with monocular and binocular TR-DME, and the characteristics of patients who developed TR-DME in the first eye after cataract surgery were also compared with those who developed TR-DME in the second eye after cataract surgery. The secondary observation index represented the association between the interval of bilateral cataract surgery and the development of TR-DME in DSBCS.

### 2.5. Statistical Analyses

The data of this study were analyzed statistically using SPSS 20.0 statistical software (IBM Corp., Armonk, NY, USA). Measurement data were expressed as the mean ± standard deviation and compared using nonparametric rank-sum tests; count data were expressed as rate (%) and compared using *χ*^2^ tests. *P* < 0.05 indicated a statistically significant difference.

## 3. Results

### 3.1. Baseline Characteristics

After strict screening according to the inclusion and exclusion criteria, a total of 1553 patients (3106 eyes) were enrolled. These included 692 males (1384 eyes) and 861 females (1722 eyes). The age range was 45 to 94 years, with an average age of 69.27 ± 9.24 years. The course of DM was 1–50 years, with an average of 9.25 ± 7.01 years. The DR stages of the patients were 1352 cases of unremarkable DR, 198 cases of NPDR, and 3 cases of proliferative diabetic retinopathy (PDR). The basic data and characteristics of the patients in the study group are shown in Table 1.

### 3.2. Rate of TR-DME Development after Bilateral Cataract Surgery

**Figure 1:**
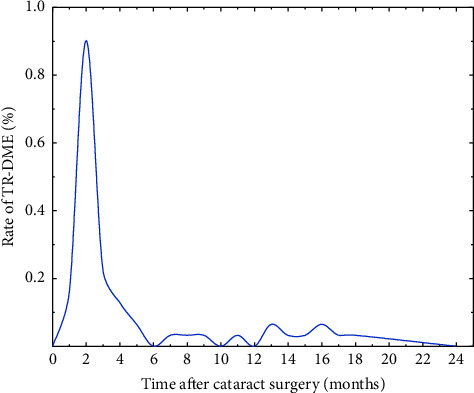
Incidence of first episode of TR-DME after bilateral cataract surgery.

In this study, there were 11 patients (16 eyes) who experienced TR-DME within two years before cataract surgery, with an incidence rate of 0.52%. Thirty-five patients (58 eyes) experienced TR-DME within two years after cataract surgery, with an incidence of 1.87%. The increase in the incidence rate of TR-DME after cataract surgery compared with the preoperative period was significant (*P* < 0.05).  Figure 1 shows the time of the first appearance of TR-DME after surgery. As observed, the highest incidence of TR-DME was recorded in the second month after cataract surgery, accounting for 41.9% of all patients with TR-DME. The period showing the highest incidence of TR-DME was between two and four months after the cataract surgery, after which the incidence of TR-DME decreased and leveled off.

### 3.3. Risk Factors in Patients Developing TR-DME after Bilateral Cataract Surgery

Table 2 shows the characteristics of patients with and without TR-DME. After comparison, statistically significant differences were observed in age, Diabetic Retinopathy Severity Scale (DRSS) scores, course of DM, and previous history of anti-VEGF injections between the two groups (*P* < 0.05), suggesting that the development of postoperative DME was closely associated with both patient characteristics and conditions of the fundus.

### 3.4. Influence of the Physician on the Interval between Bilateral Cataract Surgery

**Figure 2:**
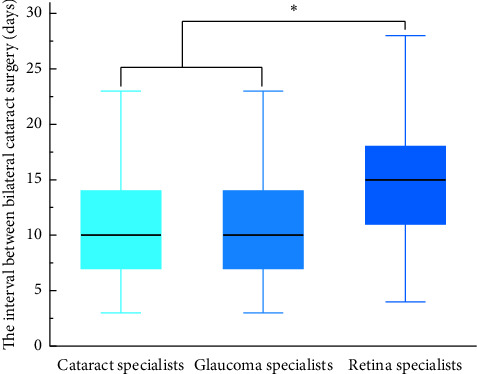
Intervals between bilateral cataract surgeries according to the ophthalmic specialty of the physicians. Note: ^*∗*^denotes statistically significant *P*-value.

The DSBCS interval was analyzed to explore the decision-making of different physicians performing cataract surgery on the interval of bilateral cataract surgery (see Figure 2). When the physician performing the second bilateral cataract surgery was different, the ophthalmic subspecialty of the second physician was used in the analysis. Overall, cataract experts performed 1303 of the bilateral cataract surgeries, with an interval of 10.69 ± 4.10 days between the procedures; glaucoma experts performed 102 operations with an interval between the procedures of 11.17 ± 5.06 days; experts in fundus disease performed 148 operations with an interval of 14.97 ± 5.36 days. There was no statistically significant difference in the intervals of bilateral cataract surgery between cataract and glaucoma experts, but both were significantly less than those performed by the fundus disease experts (*P* < 0.05).

### 3.5. Incidence of TR-DME after Sequential Bilateral Cataract Surgery

All patients who underwent DSBCS were divided into four groups according to the interval of bilateral cataract surgery (1–4 weeks) to compare differences in the occurrence of TR-DME after surgery (see Table 3). The number of days between the bilateral sequential cataract surgeries was converted to weeks for statistical analysis. The results showed a significant difference in the incidence rate of TR-DME between patients with a two-week interval between bilateral cataract surgery and those with a three-week interval, and the incidence rate of TR-DME in patients with intervals ≤2 weeks differed significantly from those with intervals of >2 weeks (*P* < 0.05) (see Table 4).

### 3.6. Incidence of Monocular/Binocular TR-DME after Sequential Bilateral Cataract Surgery

Overall, 23 patients (65.7%) developed TR-DME in binocular eyes after bilateral cataract surgery, and 12 patients (34.2%) developed monocular TR-DME. Information on the association between the incidence of monocular or binocular TR-DME and patient characteristics is shown in Table 5. It was observed that among all patients with TR-DME, women were more likely to develop monocular TR-DME (*P* < 0.05).

### 3.7. Incidence of First/Second Eye TR-DME after Sequential Bilateral Cataract Surgery

Twenty-three patients (65.7%) developed TR-DME in the first eye after surgery, with 12 patients (34.2%) developing TR-DME in the second eye. Details of the associations between TR-DME development in the first or second eyes, patient characteristics, and intervals between surgeries are shown in Table 6. No statistically significant correlations were seen between age, DRSS, or interval between cataract surgeries in both eyes, nor between the eyes that developed TR-DME first after surgery.

## 4. Discussion

Common complications after diabetic cataract surgery include posterior capsular opacification, DME, and endophthalmitis [16]. DME is the main cause of visual impairment in DM patients after cataract surgery. The mechanism underlying the development of DME after cataract surgery is complex and involves multiple factors. The main mechanisms include disruption of the blood-retinal barrier, massive release of VEGF and inflammatory factors, and changes in the vitreo-retinal interface. Additionally, longer ultrasound usage during cataract surgery and higher energy consumption may also cause or aggravate DME. A large cohort study that included two independent surgical centers in China and the United States investigated the risk factors affecting CDE levels in cataract surgery. In the multivariable analysis, diabetes, the age of the patient, and the skill and proficiency of the surgeon were found to be significant factors [17].

This single-center study collected data from hospitalized DC patients from an eye hospital in Northwestern China over a three-year period. These real-world data showed that the incidence rate of TR-DME after bilateral cataract surgery with a first occurrence rate of 1.87%. In the literature, the reported incidence rate of DME tends to vary according to study design, sample size, definition, and classification of DME. A retrospective study of 5380 eyes treated with different postoperative eye-drop regimens observed an overall incidence of macular cystoid edema of 0.82% at 1–3 months after surgery [18]. However, in another multicenter observational study, 51.8% of DM patients exhibited different forms of macular edema after cataract surgery [19]. Howaidy et al. [20] reported that 3.45% (representing four patients) of patients with macular edema required further vitreous injection of ranibizumab for treatment. Unlike the definition of postoperative DME in other studies, the present study conformed to the criteria used in a large sample retrospective study in the UK and adopted their definition of TR-DME, namely, a serious type of DME that was considered by the consulting physician to require vitreous anti-VEGF injection after evaluation of the patient's visual acuity and fundus OCT examination. The diagnosis of DME thus relied on the physician's judgment based on the actual condition of the patient and the fundus imaging results, which was more subjective but closer to the distribution and treatment of DME in clinical practice in real-world situations.

A retrospective study [21] showed that the incidence of macular edema increased in patients with different degrees of DR (from 25% to 46.2%) one year after cataract surgery, while the number of patients that ultimately received vitreous injections was very low, accounting for 8.7% of the total. Injection administration was higher in the group with more severe DRSS. In our study, 13.6% of patients with NPDR developed TR-DME, and in the group with more severe PDR, all patients developed TR-DME. Our findings are consistent with those of an earlier multicenter study [22], in which patients without preoperative DR had an increased risk of DME after cataract surgery (OR 1.844). The risk of postoperative DME increased in patients with DR over the range of mild NPDR (OR 2.375) to moderate NPDR (OR 6.556), and to severe NPDR (OR 7.428). Furthermore, while PDR had a significant effect on postoperative edema (OR 4.031), it was lower than the OR values of moderate and severe NPDR.

Even in the absence of significant preoperative DR, 0.37% of patients without DR developed TR-DME after surgery. In this study, the interval between the surgeries was ≤2 weeks in five patients, while the incidence of TR-DME in patients with surgical intervals ≤2 weeks differed significantly from that in patients with surgical intervals >2 weeks. To the best of our knowledge, this is the first description of the relationship between bilateral cataract surgery and the development of TR-DME in patients with DSBCS. Through the detection of cytokine levels in aqueous cells in different eye diseases, Tang et al. [14] found that the concentrations of various inflammatory cytokines in the aqueous humor of DM patients were higher before surgery, while the levels of intraocular IL-2 and VEGF increased after cataract surgery. Based on the above studies, it is hypothesized that a shorter interval between surgeries may exacerbate the expression of inflammatory factors and cytokines, such as VEGF, in the eyes of DM patients, leading to the development of DME.

A comparison of aqueous cell cytokine levels in the first and second TR-DME episodes after DSBCS was reported previously [23], and the results showed that TGF-*β* levels were higher in the second eye compared with the first, suggesting that the intraocular microenvironment of the contralateral eye was altered by the first cataract surgery. Zhang et al. [24] demonstrated that patients with bilateral cataract experienced higher levels of pain in the second eye compared with the first eye, and the concentration of the chemokine monocyte chemoattractant protein-1 (MCP-1) in the aqueous humor of the second eye was increased. In the present study, different intervals between cataract surgeries were compared with the incidence rate of postoperative single/bilateral eye and first/second episode of TR-DME, and the results showed that patients with a surgical interval of ≤2 weeks showed greater occurrences of both binocular TR-DME and first-episode TR-DME compared with monocular TR-DME and the second-episode TR-DME, although the differences were not significant. This may be related to the fact that DR staging was not included in the present study for comparison, and the influence of factors was confounded.

The similarity of binocular vision results in binocular overlap, which improves patient satisfaction and functional vision in eyes with binocular intraocular lenses (IOL). Therefore, bilateral cataract surgery should be performed in a timely manner when cataract occurs in both eyes. However, there are no accurate data on the interval between bilateral cataract surgeries either in China or abroad. It is often thought that, due to possibilities such as endophthalmitis, anterior segment toxicity syndrome, and refractive error, the second eye can be operated on when the first eye has reached a medically stable state after cataract surgery, and physicians usually choose to perform the second eye surgery four weeks after cataract surgery on the first eye. However, in the real world, the interval varies from surgeon to surgeon. In this study, even in the same hospital, different physicians were found to have made different decisions regarding the interval of bilateral cataract surgery. This may be related to the hospital's registration and triage system, where patients with longstanding DM and poor fundus conditions are more likely to seek medical attention from fundus disease specialists. The results of this study suggest that for some patients with high risk factors for DC, a longer interval before performing cataract surgery on the second eye should be considered to fully observe the progression of DR before performing cataract surgery.

This study also has some limitations and shortcomings. Since the data source was the hospital EMR system, the diagnosis of TR-DME was based on diagnostic and surgical records and lacked specific results from preoperative and postoperative OCT. Therefore, it was difficult to distinguish the possibility of postoperative Irvine–Gass syndrome. However, as the definition states, all patients were treated with vitreous anti-VEGF injections. In China, treatment of Irvine–Gass syndrome usually involves nonsteroidal anti-inflammatory or hormonal drops as the first-line treatment, while vitreous anti-VEGF drops are the second line of treatment; the latter accounts for a very small proportion of the actual clinical practice. At the same time, all such procedures must be preceded by OCT or OCTA examination before surgery, representing an indirect reflection of the severity of the macular condition.

The diagnosis and classification of DR do not meet the same criteria for a specific classification, and there is a lack of specific values for preoperative blood glucose, hemoglobin A1C (HbA1C), and blood lipid levels. Although Denier et al. [25] claimed that there was no linear correlation between HbA1C levels and central retinal thickness (CRT), Bar-Oz [26] found that DR stage and HbA1C were the main factors influencing the development of DME in patients after cataract surgery. Therefore, the correlation between the interval of bilateral cataract surgery and TR-DME needs to be further investigated.

This study was a real-world retrospective study and thus involved issues such as medical insurance policies and patients' medical treatment in different places in China. It is thus possible that the incidence of postoperative TR-DME observed in this study may have been lower than the actual DME incidence. Although not as standard as randomized controlled trials with standardized experimental designs and accurate follow-up data, real-world studies often reflect the clinical treatment situation. The present study found that the highest incidence of TR-DME occurred between two and four months following cataract surgery. In the northwestern region of China, cataract surgery follow-ups are conventionally conducted 1 day, 1 week, 1 month, 3 months, 6 months, and 1 year after the surgery. In actual clinical practice, however, not all patients are treated with cataract surgery and not all patients can be followed up at the predetermined times, and doctors may not review OCT to assess DME at each follow-up. In this study, two months after surgery showed the highest incidence of TR-DME, which is not a routine follow-up time point. Therefore, the findings of this study may provide a reference for China regarding the follow-up time and frequency of postoperative OCT.

In summary, this study collected data on DM patients who underwent bilateral cataract surgery from a single center in Northwestern China and analyzed the relationship between the incidence rate of TR-DME and the interval of bilateral cataract surgery. These real-world data suggest that the incidence rate of DME in DM patients was significantly increased following cataract surgery. In clinical practice, the interval between bilateral cataract surgeries should be appropriately extended for DM patients with bilateral cataracts, especially in the case of older patients and those who have had diabetes for longer. Furthermore, the study findings also provide a reference for the follow-up of DM patients. In the early postoperative period, even for patients with mild DR, regular OCT examinations should be performed, allowing the detection and treatment of DME at an early stage, to maintain good visual quality.

## Figures and Tables

**Table 1:** Baseline demographic and clinical characteristics of the study sample.

Variable	*N* = 1553
Age, years	
Mean	69.27
Range	45–94
Sex, *n* (%)	
Female	692 (44.6%)
Male	861 (55.4%)
Diabetic retinopathy, *n* (%)	
No	1352 (87.05)
Nonproliferative	198 (12.75)
Proliferative	3 (0.19)
Length of DM, years	
Mean	9.25
Range	1–50

**Table 2:** Patient characteristics associated with the development or nondevelopment of TR-DME.

	No TR-DME	TR-DME	*P* value
Age (years)	69.42 ± 9.20	63.17 ± 9.29	<0.001^*∗*^
DRSS			
No DR	1347	5	<0.001^*∗*^
NPDR	171	27	
PDR	0	3	
Length of DM (years)	9.16 ± 6.98	12.99 ± 7.35	0.003^*∗*^
History of anti-VEGF injections	8/1518 = 0.53%	3/35 = 8.57%	<0.001^*∗*^

Note.^*∗*^denotes statistically significant *P* value.

**Table 3:** Incidence of TR-DME in relation to different intervals between sequential bilateral cataract surgeries.

Interval between bilateral cataract surgery	Without TR-DME (*n* = 3036 eyes)	With TR-DME (*n* = 58 eyes)
1 week (1–7 days)	890	13
2 weeks (8–14 days)	1674	42
3 weeks (15–21 days)	396	1
4 weeks (22–31 days)	76	2

**Table 4:** Pairwise comparisons of TR-DME incidence and interval between sequential bilateral cataract surgeries.

Pairwise comparison	*χ* ^2^	*P* value
1 week-2 weeks	2.923	0.087
1 week–3 weeks	2.622	0.105
1 week–4 weeks	0.087	0.768
2 weeks-3 weeks	7.797	0.005^*∗*^
2 weeks–4 weeks	0.000	1.000
3 weeks-4 weeks	2.480	0.115
0–14 days–15–21 days	6.642	0.011^*∗*^
0–14 days–22–31 days	4.713	0.030^*∗*^

Note. ^*∗*^denotes statistically significant *P* value.

**Table 5:** Incidence of monocular/binocular TR-DME after sequential bilateral cataract surgery.

	Monocular TR-DME (*n* = 12)	Binocular TR-DME (*n* = 23)	*P* value
Age (years)	62.67 ± 10.21	63.43 ± 9.00	0.820
Sex			
M	2	15	0.006^*∗*^
F	10	8	
DRSS			
No DR (cases)	3	2	0.142
NPDR (cases)	9	18	
PDR (cases)	0	3	
Course of DM (years)	12.50 ± 9.19	13.30 ± 6.27	0.761
Interval of bilateral cataract surgery (days)	12.83 ± 2.89	11.13 ± 3.71	0.061

Note.^*∗*^denotes statistically significant *P* value.

**Table 6:** Incidence of first/second episode of TR-DME after sequential bilateral cataract surgery.

	First episode of TR-DME (*n* = 23)	Second episode of TR-DME (*n* = 12)	*P* value
Age (years)	61.48 ± 9.17	66.42 ± 9.01	0.138
Sex			
M	11	6	0.903
F	12	6	
DRSS			
No DR (cases)	4	1	0.174
NPDR (cases)	16	11	
PDR (cases)	3	0	
Course of DM (years)	13.02 ± 7.27	13.00 ± 7.69	0.972
Interval of bilateral cataract surgery (days)	11.57 ± 3. 00	12.00 ± 4.45	1.000

## Data Availability

The datasets used and/or analyzed during the current study are available from the authors upon reasonable request.
